# Correction: Unified pre- and postsynaptic long-term plasticity enables reliable and flexible learning

**DOI:** 10.7554/eLife.11988

**Published:** 2015-10-09

**Authors:** Rui Ponte Costa, Robert C Froemke, P Jesper Sjöström, Mark CW van Rossum

Costa PR, Froemke RC, Sjöström PJ, van Rossum MCW. 2015. Unified pre- and postsynaptic long-term plasticity enables reliable and flexible learning. *eLife* **4**:09457. doi: 10.7554/eLife.09457

Published 26 August 2015

In the originally published article, the affiliations for Rui Ponte Costa and Robert C Froemke were given incorrectly.

Rui Ponte Costa's fourth affiliation was indicated as being the Skirball Institute for Biomolecular Medicine, Departments of Otolaryngology, Neuroscience and Physiology, New York University School of Medicine, New York, United States. The fourth affiliation should have been the Centre for Neural Circuits and Behaviour, University of Oxford, Oxford, United Kingdom.

Robert C Froemke was wrongly indicated as being affiliated with the Centre for Neural Circuits and Behaviour, University of Oxford, Oxford, United Kingdom. He should have been shown to be affiliated with both the Skirball Institute for Biomolecular Medicine, Departments of Otolaryngology, Neuroscience and Physiology, New York University School of Medicine, New York, United States, and the Center for Neural Science, New York University, New York, United States.

The article has now been corrected.

