# Correction: Unified pre- and postsynaptic long-term plasticity enables reliable and flexible learning

**DOI:** 10.7554/eLife.108860

**Published:** 2025-08-21

**Authors:** Rui Ponte Costa, Robert C Froemke, Per Jesper Sjöström, Mark CW van Rossum

**Keywords:** Rat

Costa RP, Froemke RC, Sjöström PJ, van Rossum MCW. 2015. Unified pre- and postsynaptic long-term plasticity enables reliable and flexible learning. *eLife* **4**:e09457. doi: 10.7554/eLife.09457.

Published 26 August 2015

There was a typo in the code used to fit the visual cortex data. Iiro Ahokainen identified this and implemented a correct implementation, which gives the updated parameters given in new Table 1. Note that these parameters give virtually the same results given in Figure 1. Code for this implementation is provided at https://github.com/IiroAhokainen/ModelFittingCosta2015.

The corrected Table 1 is shown here:

**Table:** 

Parameter	*d*−	*τ_y_*_−_ (ms)	*d*+	*τ_y_*_+_ (ms)	*c*+	*τ_x_*_+_ (ms)
Young rat visual cortex	0.00389	12.5	0.002483	417.8	0.013706	48.6

The originally published Table 1 is shown for reference:

**Table:** 

Parameter	*d*−	*τ_y_*_−_ (ms)	*d*+	*τ_y_*_+_ (ms)	*c*+	*τ_x_*_+_ (ms)
Young rat visual cortex	0.1771	32.7	0.1548	230.2	0.0618	66.6

The article has been corrected accordingly.

